# Lipocalin 2 Does Not Play A Role in Celastrol-Mediated Reduction in Food Intake and Body Weight

**DOI:** 10.1038/s41598-019-49151-8

**Published:** 2019-09-05

**Authors:** Xudong Feng, Dongxian Guan, Thomas Auen, Jae Won Choi, Mario Andres Salazar-Hernandez, Farhana Faruk, Kyle D. Copps, Umut Ozcan

**Affiliations:** Division of Endocrinology, Boston Children’s Hospital, Harvard Medical School, Boston, Massachusetts USA

**Keywords:** Target identification, Obesity

## Abstract

Celastrol is a leptin-sensitizing agent with profound anti-obesity effects in diet-induced obese (DIO) mice. However, the genes and pathways that mediate celastrol-induced leptin sensitization have not been fully understood. By comparing the hypothalamic transcriptomes of celastrol and vehicle-treated DIO mice, we identified lipocalin-2 (*Lcn2*) as the gene most strongly upregulated by celastrol. LCN2 was previously suggested as an anorexigenic and anti-obesity agent. Celastrol increased LCN2 protein levels in hypothalamus, liver, fat, muscle, and bone marrow, as well as in the plasma. However, genetic deficiency of LCN2 altered neither the development of diet-induced obesity, nor the ability of celastrol to promote weight loss and improve obesity-associated dyshomeostasis. We conclude that LCN2 is dispensable for both high fat diet-induced obesity and its therapeutic reduction by celastrol.

## Introduction

Pervasive obesity presents a challenge to human health worldwide. In 2015, an estimated 107.7 million children, representing 5% of all children globally, were obese^1^. By 2025, 39% of adults (18% of men and 21% of women) are predicted to be obese, including 15% (6% of men and 9% of women) with over 40 kg/m^2^ body mass index (BMI)^2,3^. Alarmingly, in only 2017, high BMI was reported to account for 4.0 million deaths worldwide^1^. Despite these facts, effective non-surgical treatments for obesity have not yet been achieved in humans.

Leptin, an adipocyte-derived hormone that suppresses appetite and reduces body weight in leptin deficiency, initially raised hopes for an effective treatment for obesity^4–10^. Soon after the initial discovery of leptin, it was realized that most of the obese population has high circulating leptin concentrations and are unresponsive to exogenous leptin administration in terms of reducing food intake and body weight, which led to the notion of leptin resistance^11,12^. Development of leptin resistance, and also cytokine resistance^13,14^ in obesity provided new insights for development of obesity, as well as a potential therapeutic avenue.

We previously identified celastrol, a pentacyclic triterpene isolated from the root extracts of thunder god vine (*Tripterygium wilfordii*), as a strong leptin sensitizer and anti-obesity agent^15^. Recent publications have also confirmed these observations^16^. Oral administration of celastrol reduces the body weight of diet-induced obese (DIO) mice more than 45%, the strongest anti-obesity effect that has been reported to date, and further ameliorates insulin resistance/type-2 diabetes, nonalcoholic steatohepatitis (NASH), hypercholesterolemia, and liver damage in DIO mice^15^. Celastrol does not have anti-obesity effects in leptin receptor mutant mice (*db*/*db*), and has minimal effect in leptin-deficient (*ob*/*ob*) obesity models^15,16^. Thus, celastrol is a leptin sensitizer, and the function of celastrol depends on intact leptin signaling.

We previously documented that celastrol reduces endoplasmic reticulum (ER) stress^15^, which plays a central role in the development of leptin resistance^17–26^. However, the principal targets or pathways through which celastrol reduces ER stress and increases leptin sensitivity remain unknown. Understanding the mechanism of action of celastrol will not only provide insights into the underlying biology of leptin signaling and resistance, but will create new avenues for the development of effective therapies for obesity.

## Results

### Celastrol increases LCN2 levels

**Figure 1:**
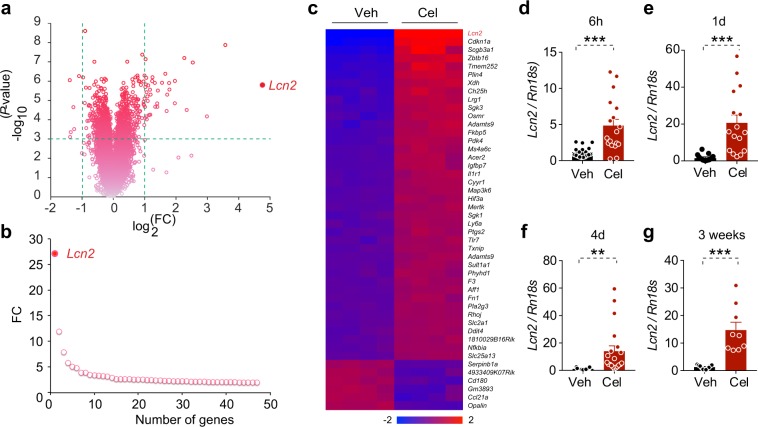
*Lcn2* is the most up-regulated gene in the hypothalamus of celastrol-treated DIO mice. (**a**) Volcano plot depicting the regulation of genes in the hypothalamus of DIO mice by celastrol. (**b**) The fold change (FC) of top celastrol-regulated genes that had FC > 2 and associated *P* < 0.001 determined by two-tailed *t* test. (**c**) Heat map representing the relative fold change of celastrol-regulated genes (40 upregulated genes and 6 down-regulated) in the hypothalamus of DIO mice treated with vehicle or celastrol (100 g/kg, i.p. daily) for 4 days. Each column represents an individual mouse, *n* = 4 mice for each group. *Lcn2* is indicated with red color. (**d**–**g**) DIO mice were treated with vehicle (Veh) or celastrol (Cel) for 6 h (250 μg/kg, i.p.), or for 1 day, 4 days or 3 weeks (100 μg/kg, once daily) and hypothalamic *Lcn2* mRNA levels were compared. Hypothalamic *Lcn2* mRNA level after (**b**) 6 h (Veh *n* = 18, Cel *n* = 18), (**c**) 1 day (Veh *n* = 15, Cel *n* = 16), (**d**) 4 days (Veh *n* = 19, Cel *n* = 20) or (**e**) 3 weeks (Veh *n* = 11, C**e**l *n* = 9). Values indicate average ± s.e.m. *P* values were determined by two-tailed Student’s *t* test. *P < 0.05, **P < 0.01, ***P < 0.001.

To investigate the mechanism underlying celastrol’s anti-obesity and leptin-sensitizing effects, we explored how hypothalamic gene expression is affected by celastrol. Diet-induced obese (DIO) mice were treated with either vehicle or celastrol for four days (100 μg/kg, i.p. injection, once daily); hypothalami were extracted and the mRNA was used for microarray hybridization as previously described^20^. To identify the top-regulated genes by celastrol, we required that two threshold criteria be simultaneously met: first, that the fold up- or down-regulation (fold change, FC) expression of each gene in celastrol-treated versus vehicle-treated mice be greater than 2 (|log_2_(FC)| > 1); and second, that the associated *P* value be less than 0.001 (-log_10_(*P*) > 3) (‘volcano’ plots in Fig. 1a). This analysis showed that celastrol treatment strongly upregulated 40 genes and down-regulated 6 genes. Lipocalin-2 (*Lcn2*) was identified as the most upregulated gene in the hypothalamus of celastrol-treated DIO mice (Fig. 1b,c).

It was previously suggested that LCN2 reduces appetite and body weight through its action on the melanocortin 4 receptor (MC4R) signaling^27^. Accordingly, we investigated whether LCN2 plays a role in celastrol-mediated reduction in food intake and body weight. We first examined the regulation of hypothalamic *Lcn2* gene expression in DIO mice treated with vehicle or celastrol for 6 hours (250 μg/kg, i.p.), or for 1 day, 4 days or 3 weeks (100 μg/kg, i.p., once daily) using quantitative PCR (qPCR). Hypothalamic *Lcn2* expression was significantly increased by celastrol at all time points tested (Fig. 1d–g).

**Figure 2:**
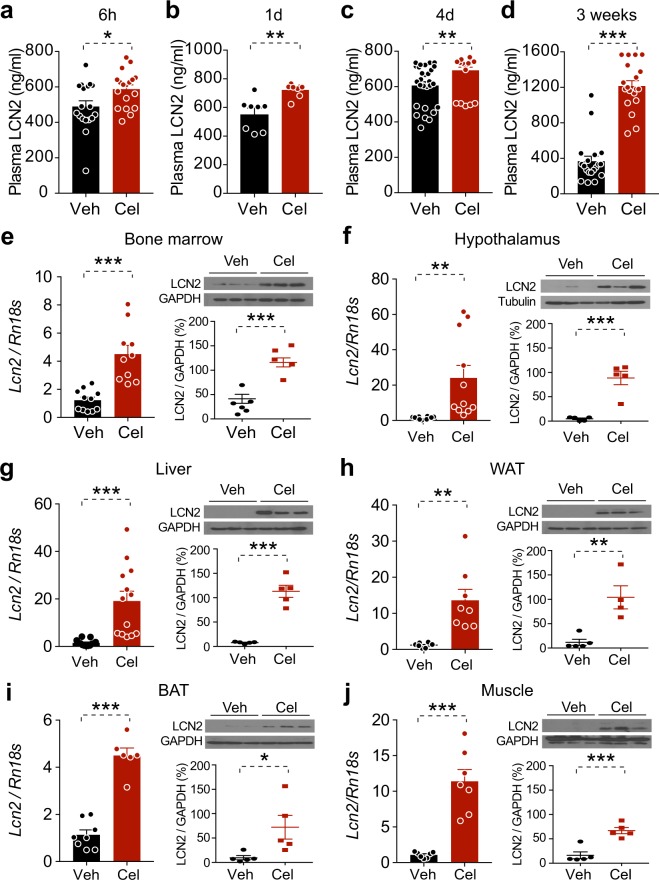
Celastrol treatment globally increases LCN2 expression. (**a**–**d**) DIO mice were administered with vehicle (Veh) or celastrol (Cel) for 6 h (250 μg/kg, i.p.), 1 day, 4 days and 3 weeks (100 μg/kg, i.p. daily) and the plasma LCN2 levels were analyzed. Plasma LCN2 levels (ng/ml) in DIO mice treated with Veh or Cel for (**a**) 6 h (Veh, *n* = 18 and Cel, *n* = 18), (**b**) 1 day (Veh, *n* = 8 and Cel *n* = 8), (**c**) 4 days (Veh, *n* = 28 and Cel *n* = 28) or (**d**) 3 weeks (Veh, *n* = 19 and Cel, *n* = 19). (**e**–**j**) LCN2 mRNA and protein expression levels were determined in (**e**) bone marrow, (**f**) hypothalamus, (**g**) liver, (**h**) inguinal white adipose tissue (WAT), (**i**) brown adipose tissue (BAT) and (**j**) muscle of DIO mice treated with Veh or Cel (100 μg/kg, i.p. daily) for 5 days. The mRNA results were a combination of three different cohorts for hypothalamus (Veh, *n* = 12 and Cel, *n* = 11), liver (Veh, *n* = 13 and Cel, *n* = 13) and of two cohorts for bone marrow (Veh, *n* = 11 and Cel, *n* = 10), WAT (Veh, *n* = 8 and Cel, *n* = 8), BAT (Veh, *n* = 8 and Cel, *n* = 6) and muscle (Veh, *n* = 7 and Cel, *n* = 7). The quantitation results for immunoblots were a combination of two different cohorts for bone marrow (Veh, *n* = 6 and Cel, *n* = 5), and two cohorts for hypothalamus (Veh, *n* = 5 and Cel, *n* = 5), liver (Veh, *n* = 5 and Cel, *n* = 5), WAT (Veh, *n* = 5 and Cel, *n* = 4), BAT (Veh, *n* = 5 and Cel, *n* = 5) and muscle (Veh, *n* = 5 and Cel, *n* = 5). Values indicate average ± s.e.m. *P* values were determined by two-tailed Student’s *t* test. *P < 0.05, **P < 0.01, ***P < 0.001.

As secretion of LCN2 from bone marrow to plasma was supposed previously to act centrally to regulate food consumption and body weight^27^, we examined whether circulating LCN2 levels were elevated by celastrol treatment. DIO mice were treated with celastrol for 6 hours (250 μg/kg) or 1 day, 4 days and 3 weeks (100 μg/kg, i.p. injection, once daily), after which plasma LCN2 was measured. Circulating LCN2 was significantly increased 6 hours after celastrol treatment (*P* < 0.05, Veh versus Cel) (Fig. 2a), and was maintained at significantly higher levels over three weeks in celastrol-treated versus vehicle-treated DIO mice (*P* < 0.01 after 1 day or 4 day treatment and *P* < 0.001 after 3 weeks treatment, Veh versus Cel) (Fig. 2b–d).

We next determined whether LCN2 in bone marrow was affected by celastrol. DIO mice were treated with vehicle or celastrol (100 μg/kg, i.p., once a day) for five days and *Lcn2* gene expression and protein levels in the bone marrow were analyzed. Celastrol administration led to significant upregulation of *Lcn2* gene expression, and also of LCN2 protein levels in the bone marrow (*P* < 0.001, Veh versus Cel) (Fig. 2e). Furthermore, analysis of gene expression and LCN2 protein levels in hypothalamus (Fig. 2f), liver (Fig. 2g), white adipose tissue (WAT) (Fig. 2h), brown adipose tissue (BAT) (Fig. 2i) and muscle (Fig. 2j) revealed a celastrol-induced increase in LCN2 levels in each of these tissues, indicating global upregulation of LCN2 by celastrol.

### LCN2 deficiency does not accelerate diet-induced obesity

**Figure 3:**
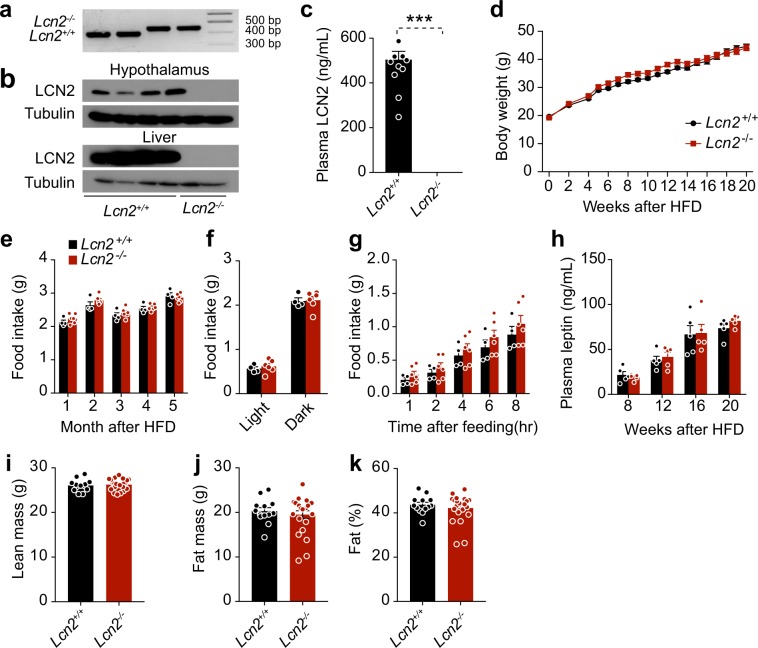
LCN2 deficiency does not accelerate diet-induced obesity. *Lcn2* knockout (*Lcn2*^−/−^) mice and their wild-type (*Lcn2*^+/+^) littermates were fed with HFD for ~20 weeks to induce obesity. Body weight and food intake were measured. (**a**) Representative image showing PCR genotyping of *Lcn2*^−/−^ and *Lcn2*^+/+^ mice. (**b**) Representative immunoblots for LCN2 and tubulin proteins in hypothalamus and liver of *Lcn2*^+/+^ and *Lcn2*^−/−^ mice after celastrol treatment (100 μg/kg, i.p., once daily) for 3 weeks. (**c**) Plasma LCN2 levels in the *Lcn2*^+/+^ and *Lcn2*^−/−^ mice after 16 weeks HFD feeding. (**d**) Body weight change of *Lcn2*^+/+^ and *Lcn2*^−/−^ mice during HFD feeding (*Lcn2*^+/+^*, n* = 13 and *Lcn2*^−/−^*, n* = 23). (**e**) Average 24-hour food intake per mouse. The experiments in d and e were repeated in two cohorts (total *n* = 25 in *Lcn2*^+/+^ group and *n* = 30 in *Lcn2*^−/−^ group). (**f**) Light and dark cycle food intake of *Lcn2*^+/+^ and *Lcn2*^−/−^ mice during 10–14 weeks of HFD feeding. (**g**) Cumulative food intake of *Lcn2*^+/+^ and *Lcn2*^−/−^ mice during 1, 2, 4, 6 and 8 hours of refeeding after 15 hours of fasting. (**h**) Plasma leptin levels of *Lcn2*^+/+^ and *Lcn2*^−/−^ mice at different weeks of HFD feeding. *n* = 5 for each group. (**i–k**) DEXA of *Lcn2*^+/+^ and *Lcn2*^−/−^ mice after 20 weeks of HFD-feeding (*Lcn2*^+/+^*, n* = 13 and *Lcn2*^−/−^*, n* = 23): (**i**) lean mass, (**j**) fat mass and (**k**) percent body fat mass after 20 weeks of HFD-feeding. Values indicate average ± s.e.m. *P* values were determined by two-way ANOVA with Bonferroni’s multiple comparisons test in d or two-tailed Student’s *t* test (c and e–k). ***P < 0.001.

The above results, along with the previous report by Mosialou *et al*.^27^, led us to hypothesize that celastrol-induced upregulation of LCN2 in the hypothalamus or circulation might mediate, or at least contribute to, the anorexigenic and anti-obesity effects of celastrol. Thus, we obtained lipocalin-2 knockout mice (*Lcn2*^−/−^)^28^ to test this hypothesis. Genomic PCR and assessment of tissue LCN2 protein were used to validate the genotype of *Lcn2*^−/−^ mice and wild-type littermates (*Lcn2*^+/+^) (Fig. 3a,b). *Lcn2*^−/−^ mice did not have detectable expression of LCN2 in different tissues, nor in plasma (Fig. 3c). When fed a HFD for 20 weeks to induce obesity, *Lcn2*^+/+^ and *Lcn2*^−/−^ mice showed equivalent body weight gain (Fig. 3d). Analysis of 24 h food intake during the course of HFD-feeding (Fig. 3e), during 12 h light or dark cycles (Fig. 3f), or during re-feeding after 16 hours of fasting (Fig. 3g**)** revealed no significant differences between the *Lcn2*^+/+^ and *Lcn2*^−/−^ mice. In keeping with these observations, we detected no significant differences in circulating leptin levels between the *Lcn2*^+/+^ and *Lcn2*^−/−^ mice (Fig. 3h**)**.

We next used dual-energy X-ray absorptiometry (DEXA) scans to determine the amount of lean and fat mass in *Lcn2*^+/+^ versus *Lcn2*^−/−^ mice. Consistent with the trend in body weight, no differences were noted in body composition, including in lean mass (Fig. 3i**)**, fat mass (Fig. 3j**)**, and percent body fat mass (Fig. 3k**)** between *Lcn2*^+/+^ and *Lcn2*^−/−^ mice after 20 weeks of HFD feeding. Together, these results indicate that LCN2 deficiency did not increase food consumption or degree of obesity during high fat diet-feeding.

### LCN2 deficiency does not alter impaired glucose homeostasis or hepatic function in DIO mice

**Figure 4:**
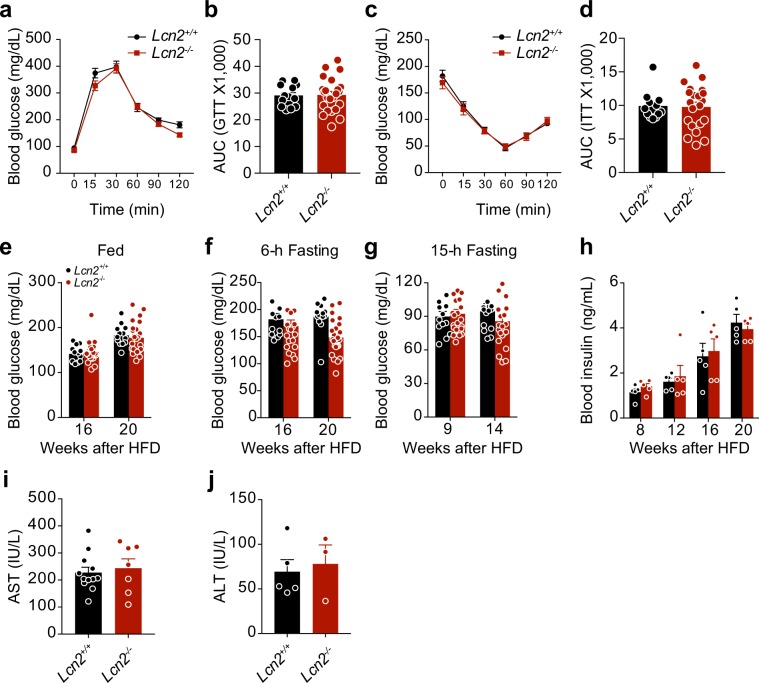
LCN2 deficiency does not alter impaired glucose homeostasis or hepatic function in DIO mice. *Lcn2* knockout (*Lcn2*^−/−^) mice and their wild-type (*Lcn2*^+/+^) littermates were fed with a HFD for ~20 weeks to induce obesity. (**a**) Glucose tolerance test (GTT) at 14 weeks of HFD-feeding, and (**b**) the area under the curve (AUC) analysis of GTT graph. (**c**) Insulin tolerance test (ITT) after 16 weeks of HFD-feeding, and (**d**) the AUC analysis of ITT graph (*Lcn2*^+/+^, *n* = 13 and *Lcn2*^−/−^, *n* = 23). (**e–g**) Fed, 6-hour fasting and 15-hour fasting blood glucose levels at indicated time points during HFD-feeding. (**h**) Plasma insulin concentrations in *Lcn2*^+/+^ and *Lcn2*^−/−^ mice at indicated time points throughout HFD-feeding. (**i**) Plasma aspartate transaminase (AST) and (**j**) alanine transaminase (ALT) levels after 16 weeks of HFD-feeding (AST, *n* = 12 for *Lcn2*^+/+^ and *n* = 7 for *Lcn2*^−/−^; ALT, *n* = 5 for *Lcn2*^+/+^ and *n* = 3 for *Lcn2*^−/−^). Values indicate average ± s.e.m. *P* values were determined by two-way ANOVA with Bonferroni’s multiple comparisons test (**a** and **c**) or two-tailed Student’s *t* test (b and d–j).

To assess the effect of LCN2 deficiency on glucose homeostasis, we performed glucose tolerance (GTT) and insulin tolerance (ITT) tests. Clearance of glucose from the circulation during GTT was similar in DIO *Lcn2*^+/+^ and *Lcn2*^−/−^ mice (Fig. 4a,b), and ITT suggested no differences in insulin sensitivity between the two groups (Fig. 4c,d). Analysis of blood glucose levels in fed versus fasted states showed no difference between the control and *Lcn2*^−/−^ mice throughout the study period (Fig. 4e,f,g). Nor did plasma insulin levels differ significantly during the 20-week HFD-feeding study (Fig. 4h). Plasma levels of aspartate aminotransferase (AST) and alanine transaminase (ALT) were similar in DIO *Lcn2*^+/+^ and *Lcn2*^−/−^ mice, indicating that LCN2 deficiency also had no effect upon liver function (Fig. 4i,j).

### LCN2 does not play a role in mediating celastrol’s anti-obesity effect

**Figure 5:**
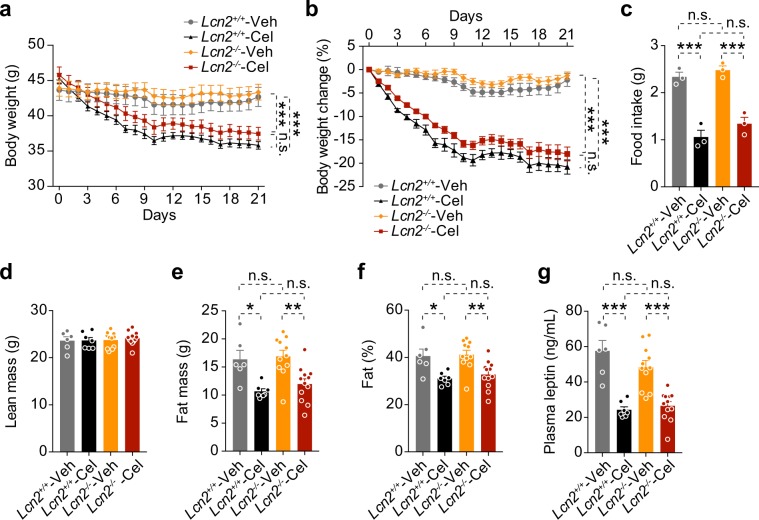
LCN2 does not mediate celastrol’s anti-obesity effect. *Lcn2*^+/+^ and *Lcn2*^−/−^ DIO mice were administrated vehicle (Veh) or celastrol (Cel, 100 μg/kg, i.p., once daily) for 3 weeks. (**a**) Body weight and (**b**) percent change in body weight of *Lcn2*^+/+^ and *Lcn2*^−/−^ mice during celastrol treatment (*n* = 6 for vehicle and *n* = 7 for celastrol in *Lcn2*^+/+^ mice; *n* = 11 for vehicle and *n* = 12 for celastrol in *Lcn2*^−/−^ mice). (**c**) Average 24-hour food intake of *Lcn2*^+/+^ and *Lcn2*^−/−^ mice during the first week of celastrol treatment. (**d–f**) DEXA after 3 weeks of celastrol treatment: (**d**) Lean mass, (**e**) fat mass and (**f**) percent body fat mass. (**g**) Plasma leptin levels (ng/ml) in *Lcn2*^+/+^ and *Lcn2*^−/−^ mice after 1 week of celastrol treatment. The experiments were repeated in two independent cohorts (total *n* = 6 for vehicle and *n* = 18 for celastrol in *Lcn2*^+/+^ group; *n* = 11 for vehicle and *n* = 19 for celastrol in *Lcn2*^−/−^ group). Values indicate average ± s.e.m. *P* values were determined by two-way ANOVA with Bonferroni’s multiple comparisons test. * *P* < 0.05, ** *P* < 0.01, *** *P* < 0.001, n.s. not significant (*P* > 0.05).

The above analyses of *Lcn2*^+/+^ and *Lcn2*^−/−^ mice found no differences in HFD-induced weight gain or associated metabolic dyshomeostasis. However, LCN2 has been implicated in reducing appetite and body weight^27,29^, and our data document that celastrol increases LCN2 levels in the circulation, as well as diverse tissues. We thus reasoned that LCN2 might play a role in mediating celastrol’s anorexigenic and anti-obesity effect, such that LCN2 deficiency would reduce celastrol’s anorexigenic effects, and blunt the ability of celastrol to reduce body weight in DIO mice. To test this hypothesis, we induced obesity in *Lcn2*^+/+^ and *Lcn2*^−/−^ mice by HFD-feeding for 20 weeks, and then treated each group with vehicle or celastrol (100 μg/kg, i.p., once a day) for three weeks. As expected, celastrol treatment led to a significant reduction in the body weight of *Lcn2*^+/+^ mice, relative to vehicle-injected *Lcn2*^+/+^ mice (*P* < 0.001, Veh versus Cel) (Fig. 5a,b). However, in two different cohorts, treatment of *Lcn2*^−/−^ mice with celastrol yielded a reduction in body weight similar to that seen in celastrol-treated *Lcn2*^+/+^ mice (*P* < 0.001, Veh versus Cel) (Fig. 5a,b and Supplementary Fig. 1a,b). Reduction in food intake in *Lcn2*^+/+^ and *Lcn2*^−/−^ mice after celastrol treatment was also not significantly different (Fig. 5c and Supplementary Fig. 1c). In addition, analysis of the total lean mass, fat mass and percent body fat mass showed that lean mass was unaltered, and percent body fat mass was reduced, to the same extent, in celastrol-treated *Lcn2*^+/+^ and *Lcn2*^−/−^ mice (Fig. 5d-f and Supplementary Fig. 1d-f) (*P* < 0.001, Veh versus Cel). In parallel, leptin levels were equivalently reduced in both groups by celastrol treatment (Fig. 5g and Supplementary Fig. 1g).

### LCN2 deficiency does not affect the improvement of glucose homeostasis by celastrol

**Figure 6:**
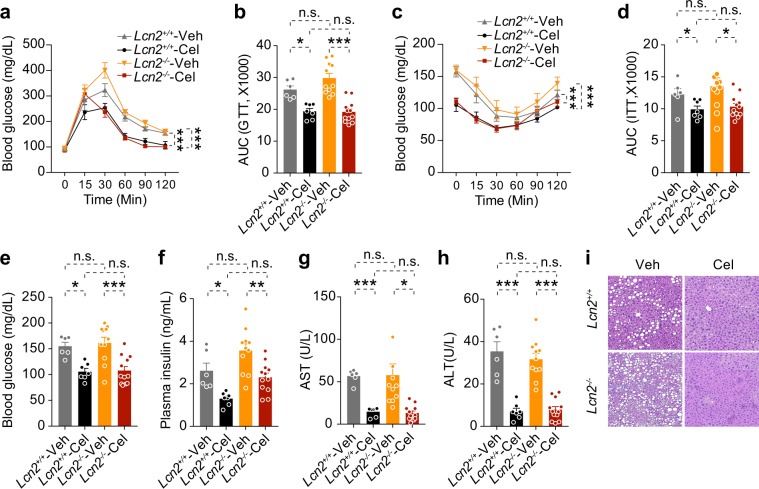
LCN2 deficiency does not affect the improvement of glucose homeostasis by celastrol. *Lcn2*^+/+^ and *Lcn2*^−/−^ DIO mice were administrated vehicle (Veh) or celastrol (Cel, 100 μg/kg, i.p., once daily) for 3 weeks. (**a**) GTT and (**b**) AUC analysis of GTT after 1 week celastrol treatment. (**c**) ITT and (**d**) AUC analysis of ITT after 2 weeks of celastrol treatment. (**e**) 6-hour fasting blood glucose after 1 week of celastrol treatment. (**f**) Plasma insulin levels after 3 weeks of celastrol treatment. Plasma (**g**) AST and (**h**) ALT levels after 3 weeks of celastrol treatment. (**i**) Hematoxylin and eosin (H&E) staining of liver sections of *Lcn2*^+/+^ and *Lcn2*^−/−^ mice after 3 weeks of celastrol treatment. Values indicate average ± s.e.m. The experiments in a-h were repeated in two independent cohorts (total *n* = 6 for vehicle and *n* = 18 for celastrol in *Lcn2*^+/+^ group; *n* = 11 for vehicle and *n* = 19 for celastrol in *Lcn2*^−/−^ group). *P* values were determined by two-way ANOVA with Bonferroni’s multiple comparisons test. *P < 0.05, **P < 0.01, ***P < 0.001, n.s. not significant (*P* > 0.05).

Celastrol treatment improves glucose homeostasis on DIO mice^15,16^. Thus, we performed GTT and ITT to investigate whether the improvement of glucose homeostasis by celastrol treatment was affected by LCN2 deficiency. GTT during the second week of treatment showed that celastrol improved glucose tolerance similarly in *Lcn2*^+/+^ and *Lcn2*^−/−^ mice (Fig. 6a,b, Supplementary Fig. 2a,b). ITT also documented that the increase in insulin sensitivity after celastrol treatment was similar in *Lcn2*^+/+^ and *Lcn2*^−/−^ mice, with no significant between-group differences (Fig. 6c,d, Supplementary Fig. 2c,d). Along with the improved glucose tolerance and insulin sensitivity, levels of blood glucose were significantly reduced in both *Lcn2*^+/+^ and *Lcn2*^−/−^ celastrol-treated mice, relative to their vehicle-treated controls (Fig. 6e, Supplementary Fig. e-g). At the end of treatment, we detected no significant differences of plasma insulin level between celastrol-treated *Lcn2*^+/+^ and *Lcn2*^−/−^ mice (Fig. 6f, Supplementary Fig. 2h). Analysis of ALT and AST levels demonstrated that celastrol improves liver function in both *Lcn2*^+/+^ and *Lcn2*^−/−^ mice (Fig. 6g,h Supplementary Fig. 2i,o). Finally, histological analysis of liver tissue revealed that celastrol reduced hepatosteatosis in both *Lcn2*^+/+^ and *Lcn2*^−/−^ mice (Fig. 6i).

**Figure 7:**
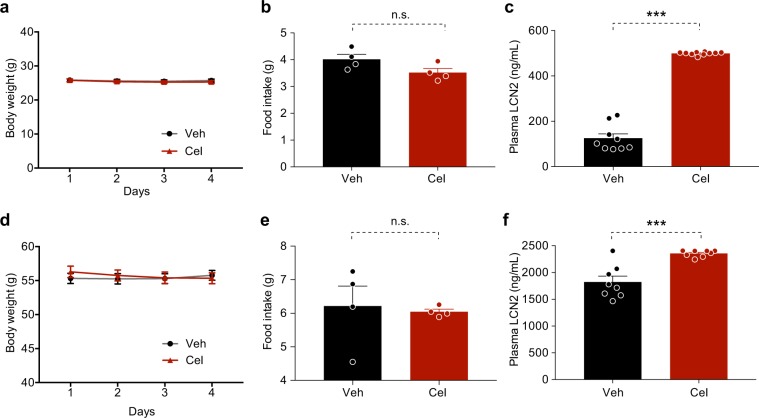
Celastrol increases plasma LCN2 levels in lean and *db*/*db* mice. Lean or *db*/*db* mice were treated with vehicle (Veh) or celastrol (Cel, 100 μg/kg, i.p., once daily) for 3 days. Body weights and food intake were measured daily. Each group of mice subsequently received a single dose of vehicle or celastrol (200 μg/kg, i.p.) in the morning of the fourth day before fasting for 6 hours. (**a**) Body weight and (**b**) average 24-hour food intake per mouse in lean mice. (**c**) Plasma LCN2 levels in lean mice after 4 days celastrol treatment. *n* = 9 for Veh and *n* = 10 for Cel. (**d**) Body weight and (**e**) average 24-hour food intake per mouse in *db/db* mice. (**f**) Plasma LCN2 levels in *db/db* mice after 4 days celastrol treatment. *n* = 8 for both groups. Values indicate average ± s.e.m. *P* values were determined by two-way ANOVA with Bonferroni’s multiple comparisons test (a,d) and two-tailed Student’s *t* test (b,c,e,f). ***P < 0.001, n.s. not significant (*P* > 0.05).

### Celastrol increases LCN2 level in celastrol-insensitive lean and *db/db* mice

Celastrol does not have an anorexigenic effect or promote weight loss in lean mice, or in leptin receptor-deficient *db*/*db* mice^15^. To investigate the relationship between LCN2 levels and celastrol responsiveness, we thus treated lean or *db*/*db* mice with celastrol (100 g/kg, i.p., once daily) for 4 days, and measured LCN2 levels in the plasma. Celastrol treatment did not induce significant body weight loss (Fig. 7a) or food intake reduction (Fig. 7b) in lean mice. However, similar to the DIO model, celastrol treatment significantly increased the LCN2 levels in the plasma of lean mice (*P* < 0.001, Veh versus Cel) (Fig. 7c). Celastrol treatment also significantly increased the plasma LCN2 levels in *db/db* mice (*P* < 0.001, Veh versus Cel) in which neither body weight nor food intake was reduced by celastrol treatment (Fig. 7d-f). Together with the normal responses of *Lcn2*^−/−^ mice to HFD feeding and celastrol (above), these results illustrate that LCN2 does not play a role in the development of diet-induced obesity, or in its therapeutic reduction by celastrol.

## Materials and Methods

### Reagents

Alanine transaminase (ALT) and aspartate transaminase (AST) color endpoint assay kits were purchased from Bio Scientific (Austin, TX). BM chemiluminescence blotting substrate (POD) and 10X blocking reagent were obtained from Roche Diagnostics, Inc. (Indianapolis, IN). Celastrol was purchased from BOC Sciences (Shirley, NY). iScript cDNA synthesis kit and SYBR Green super mix were bought from Bio-Rad (Hercules, CA). Horseradish peroxidase (HRP)-conjugated anti-goat antibody was obtained from Santa Cruz Biotechnologies (Santa Cruz, CA). GAPDH-specific antibody (#2118) and Horseradish peroxidase (HRP)-conjugated anti-rabbit antibodies were purchased from Cell Signaling Technology (Beverley, MA). Lipocalin-2/NGAL antibody (AF1857), its corresponding ELISA kit (MLCN20), and recombinant mouse leptin protein were obtained from R&D Systems (Minneapolis, MN). Mouse leptin and ultra-sensitive mouse insulin ELISA kits were purchased from Crystal Chem, Inc. (Downers Grove, IL). TRIzol reagent was obtained from Invitrogen (Carlsbad, CA). RNeasy MinElute Cleanup Kit was bought from Qiagen (#74101, Valencia, CA).

### Animals and treatment

The Animal Care and Use Committee at Boston Children’s Hospital approved all animal experiments conducted in this study (protocol number: 16-05-3168 R). All the animal experiments were conducted according to the Standard Operation Procedures (SOP) of the Institutional Animal Care and Use Committees, IACUC.

Wild-type C57BL6/J mice (stock number 000664), *db/db* mice (stock number 000697), and *Lcn2* homozygous knockout mice (stock number 024630) were purchased from Jackson Laboratories. Mice heterozygous for the *Lcn2* mutation (*Lcn2*^+/−^) were generated by crossing male *Lcn2* homozygous knockout (*Lcn2*^−/−^) mice with wild-type female mice. Wild-type (*Lcn2*^+/+^) and homozygous littermates of *Lcn2*^−/−^ mutations were generated by intercrossing *Lcn2*^+/−^ mice.

To obtain diet-induced obese (DIO) mice, wild-type C57BL6/J mice and *Lcn2*^−/−^ or *Lcn2*^+/+^ male mice were placed on high fat diet (HFD, 45 kcal% from fat; cat. D12451, Research Diets, New Brunswick, NJ) when four-weeks-old and maintained on the same diet for 16–20 weeks. Eight-week-old *db/db* and lean mice were placed on normal chow diet (NCD, 13.5% calories from fat; cat. 0006973, Lab Diets, St Louis, MO). Mice were housed in a 12-h dark/light cycle with the dark cycle lasting from 7 PM to 7 AM. All mice had *ad libitum* access to food and water unless otherwise indicated. Celastrol was dissolved in sterile DMSO (25 µl), and administered to the mice intraperitoneally (i.p) 60–90 min prior to the dark cycle, unless otherwise specified. The corresponding vehicle groups were injected i.p with a total volume of 25 µl sterile DMSO.

### Blood collection

Blood was collected from the tail vein with heparinized capillary tubes, then transferred to ice-cold eppendorf tubes and centrifuged at 3000 rpm for 30 min at 4 °C. Plasma portions were transferred to new vials and stored at −80 °C.

### Bone marrow isolation

To detect celastrol’s effect on bone marrow expression levels, DIO mice were treated with either vehicle or celastrol (100 μg/kg, i.p. daily) for four days; 60 min after the start of the light cycle on the fifth day, the mice were administered with either vehicle or celastrol (200 μg/kg, i.p.) and then fasted for 6 h. The bone marrow was flushed from femoral medullary cavities into 15 ml tubes with ice-cold PBS using a 25-gauge needle and 5 cc syringe. Cells were then centrifuged at 1,000 rpm for 10 min at 4 °C. The resulting supernatant was discarded. Bone marrow was frozen immediately in liquid nitrogen and stored at −80 °C until analysis.

Bone marrow was collected from mice in three independent cohorts. In the first cohort, bone marrow was flushed into 15 ml tubes and set on ice for 2 h before centrifugation. This allowed us to finish additional tissue collection within the cohort, but the quality of the bone marrow was affected. Thus, we excluded the results from bone marrow analysis from this cohort. In the second and third cohorts, to ensure the samples’ quality, we flushed the bone marrow into 15 ml tubes, centrifuged immediately, and froze the samples using liquid nitrogen right after the supernatant was removed.

### Body composition measurement

We assessed total lean mass, fat mass, and body fat percentage using dual-energy X-ray absorptiometry (DEXA; Lunar PIXImus2, GE Lunar Corp., Madison, WI, USA).

### Glucose tolerance test (GTT) and insulin tolerance test (ITT)

For GTT, mice were fasted for 15 h (5 PM to 8 AM) and dextrose (1 g/kg) was administered i.p. Blood glucose levels were measured from the tail before, and 15, 30, 60, 90 and 120 min after dextrose administration.

For ITT, mice were fasted for 6 h (from 8 AM to 2 PM). Recombinant human insulin (1 IU/kg) was administered i.p. Blood glucose levels were measured from the tail before, and 15, 30, 60, 90 and 120 min after insulin administration.

### Total protein extraction and western blotting

Hypothalamus, brown adipose tissue, inguinal adipose tissue, liver, fat, muscle and bone marrow were homogenized with a bench-top TissueLyser II in ice-cold tissue lysis buffer (25 mM Tris-HCl, pH 7.4; 100 mM NaF; 50 mM Na_4_P_2_O_7_; 10 mM Na_3_VO_4_; 10 mM EGTA; 10 mM EDTA; 1% NP-40; supplemented with phosphatase and protease inhibitors) and then subjected to centrifugation at 13,400 g for 30 min at 4 °C. Protein concentration was quantified using a Protein Assay Kit (Bio-Rad). Protein samples were mixed with 5x Laemmli buffer and boiled at 95 °C for 5 min before loaded onto sodium dodecyl sulfate polyacrylamide gels (SDS-PAGE). After electrophoresis, we transferred the proteins onto PVDF membranes at 4 °C, 100 V for 2 h, and blocked the membranes in TBS-0.1% Tween-20 (TBST) with 10% blocking reagent. We incubated the membranes with primary antibodies overnight in TBST containing 10% blocking reagent. After primary incubation, we washed the membranes three times for 20 min with TBST and then incubated the membranes with secondary antibodies in TBST with 10% blocking reagent for 1 h at room temperature. After washing the membrane three times in TBST, we developed the membranes using a chemiluminescence assay system and quantified band intensities using ImageJ (NIH).

### Total RNA extraction and microarray analysis

The DIO mice were acclimated to i.p. injections with vehicle (DMSO) for four consecutive days. After this acclimation period, we treated four DIO mice with vehicle and four mice with celastrol (100 µg/kg) for four days 60–90 min prior to dark cycle by i.p. injection. After 15 h, the mice were received an additional injection with either vehicle or with celastrol (200 µg/kg). Six hours later, we extracted hypothalami and froze them in liquid nitrogen. Hypothalami were stored at −80 °C until RNA extraction. To extract total RNA, we removed the hypothalami from −80 °C and added 500 µl of TRIzol to each sample. The tissues were homogenized with a bench-top TissueLyser II (Qiagen, Valencia, CA), and the hypothalamic RNA was extracted according to the manufacturer’s instruction of TRIzol lysis reagent. For microarray analysis, the extracted total RNA was cleaned using RNeasy Min Cleanup Kit (74104, QIAGEN) and 1 µg total RNA was used for microarray.

### cDNA Synthesis and quantitative real-time PCR

DIO mice were acclimated with DMSO for seven days and injected (i.p.) with DMSO or Celastrol at desired times. The tissues, including hypothalamus, brown adipose tissue, inguinal adipose tissue, liver, muscle and bone marrow, were removed 6 h after the final injections and fasting. Total RNA was extracted from the tissues with TRIzol lysis reagent following manufacturer’s protocol. Complementary DNA (cDNA) was synthesized with 1 μg of total RNA using iScript cDNA synthesis kit (Bio-Rad) according to the manufacturer’s protocol. QPCR was conducted by SYBER GREEN on QuantStudio™ 6 Flex Real-Time PCR system (Life Technologies). The relative expression of genes of interest was calculated by comparative Ct method and *RN18S* was used as an endogenous control. The primers used to detect the genes are as follows:

*Lcn2*:  Forward: 5′-TGGCCCTGAGTGTCATGTG-3′

Reverse: 5′-CTCTTGTAGCTCATAGATGGTGC-3′

*RN18S:*  Forward: 5′- AGTCCCTGCCCTTTGTACACA-3′

Reverse: 5′- CGATCCGAGGGCCTCACTA-3′

### Hormone and metabolite measurements from mouse plasma

Plasma LCN2, leptin, insulin, ALT, and AST were measured using the corresponding ELISA or assay kits according to the manufacturer’s instructions. The plasma from DIO mice was diluted 100 times in LCN2 ELISA and 5–10 times in leptin ELISA. We used 5 µL of plasma for insulin ELISA and AST assay, and 10 µL for ALT assay.

### Hematoxylin and eosin (H&E) staining

After treating DIO mice with vehicle or Celastrol (100 μg/kg) for three weeks, their livers were dissected and stored in 10% buffered formalin phosphate. Paraffin embedded liver sections were H&E stained.

### Statistical analysis

All data is presented as mean ± S.E.M. Statistical significance was measured using Student’s t-test (two-tailed) or two-way ANOVA Bonferroni’s multiple comparisons test as indicated in figure legends. *P* values below 0.05 were considered significant. Numbers of cohorts and *n* values for each experiment were indicated in figure legends. Dead or sick mice before the end of experiments or statistical outliers (judged by Grubb’s outlier test) were excluded in the final analysis. No blinding or randomization was used. No statistical method was used to pre-determine sample size and sample size was determined based on previous experiments and literature. The variance was similar in the groups being compared.

## Discussion

Celastrol, the most effective anti-obesity agent reported to date^15^, reduces ER stress, which is a central mechanism involved in the development of leptin resistance and the regulation of feeding and body weight during excess calorie consumption^17,25,26^. Thus, understanding the networks involved in celastrol’s anti-obesity effects can uncover major targets for treatment of obesity and open novel avenues for the development of obesity therapeutics. Exclusion of certain potential mediators of celastrol’s actions is also essential to this experimental goal.

LCN2 is a potent bacteriostatic agent and plays a role in the iron-depletion strategy of the immune system to control pathogens by binding to iron-loaded siderophores^30,31^. In the central nervous system (CNS), less is known about the processes involving LCN2, including by which cells it is produced/secreted, its potential impact on cell proliferation and death, or on neuronal plasticity and behavior^32^.

There are conflicting results in the literature about the phenotype of LCN2 knockout mouse models regarding obesity and metabolic homeostasis. Peripheral expression levels of LCN2, including in plasma and adipose tissue, have been reported to positively correlate with obesity^33,34^. Yan et. al. also reported that LCN2 promotes insulin resistance in isolated adipocytes and hepatocytes^34^. Compatible with these results, LCN2 deficiency was suggested to protect against HFD-induced obesity and insulin resistance^35^. However, Moialou *et al*. have reported that global deletion of *Lcn2* increases food intake and body weight under chow diet feeding^27^, and Guo et. al. have suggested that DIO *Lcn2*^−/−^ mice are prone to development of obesity and glucose intolerance^29^. We found no difference in the body weights of *Lcn2*^+/+^ and *Lcn2*^−/−^ mice. Nor did we find any difference in glucose homeostasis parameters between *Lcn2*^+/+^ and *Lcn2*^−/−^ mice. It is not uncommon to see different phenotypes from similar mouse models, or from the same mouse model in different animal facilities, and even the same mouse models maintained in the same animal facility can develop different phenotypes over time^36^. Regardless, we note that inherited, systemic LCN2 deficiency might promote metabolic adaptation in mice that we and others have studied. Thus, tissue and/or temporal-specific deletion of *Lcn2* might be a useful adjunct for integrating disparate findings regarding LCN2 function in future studies.

LCN2 expression is enriched in the bone-marrow, and secretion of LCN2 from bone marrow to plasma has been suggested to increase the MC4 receptor activity in the brain and reduce food intake and body weight^27^. Considering our own results that celastrol increases bone marrow and plasma LCN2, we hypothesized that LCN2 might mediate the anti-obesity effects of celastrol. *Lcn2*^+/+^ and *Lcn2*^−/−^ DIO mice achieved an identical degree of obesity on HFD feeding before celastrol treatment, despite the fact that knockout mice did not have any detectable LCN2. We expected that if LCN2 were an important anorexigenic factor, LCN2 deficiency during celastrol treatment should at least blunt celastrol’s appetite suppressing and anti-obesity effect at some level. However, *Lcn2*^−/−^ DIO mice responded completely normally to celastrol. Furthermore, we found that celastrol significantly increases LCN2 plasma levels in celastrol non–responsive *db*/*db* mice, wherein MC4R signaling remains intact. It would be also expected that increased circulating levels of LCN2, a putative MC4R agonist, would suppress food intake in the *db/db* mice to some degree. However, *db/db* mice treated with celastrol did not differ from vehicle-treated controls. Thus, we conclude that LCN2 is very unlikely to be a major influence on anorexigenic and anti-obesity signaling, and does not play any role in celastrol-mediated food intake and body weight reduction, despite its increased levels in the bone marrow and plasma after celastrol treatment.

## Supplementary information

Supplementary information

## Data Availability

The raw data for mouse hypothalamic microarray is available with accession number GSE84156^20^. All other data that support the findings of this study are available from the corresponding author upon request.
