# Supplementary material for: Lipocalin 2 Does Not Play A Role in Celastrol-Mediated Reduction in Food Intake and Body Weight
Source: Sci Rep. 2019 Sep 5;9:12809. doi: 10.1038/s41598-019-49151-8 (PMC6728323; doi:10.1038/s41598-019-49151-8)
Supplement: Supplementary file 1 — Supplementary information [file 41598_2019_49151_MOESM1_ESM.pdf]

Lipocalin 2 Does Not Play A Role In Celastrol-Mediated Reduction In Food Intake And Body Weight

Xudong Feng[1], Dongxian Guan[1], Thomas Auen[1], Jae Won Choi[1], Mario Andres Salazar-Hernandez[1], Farhana Faruk[1], Kyle D. Copps[1] & Umut Ozcan[1*]

[1]Division of Endocrinology, Boston Children's Hospital, Harvard Medical School, Boston, Massachusetts, USA.

[*]Correspondence should be addressed to UO

(umut.ozcan@childrens.harvard.edu)

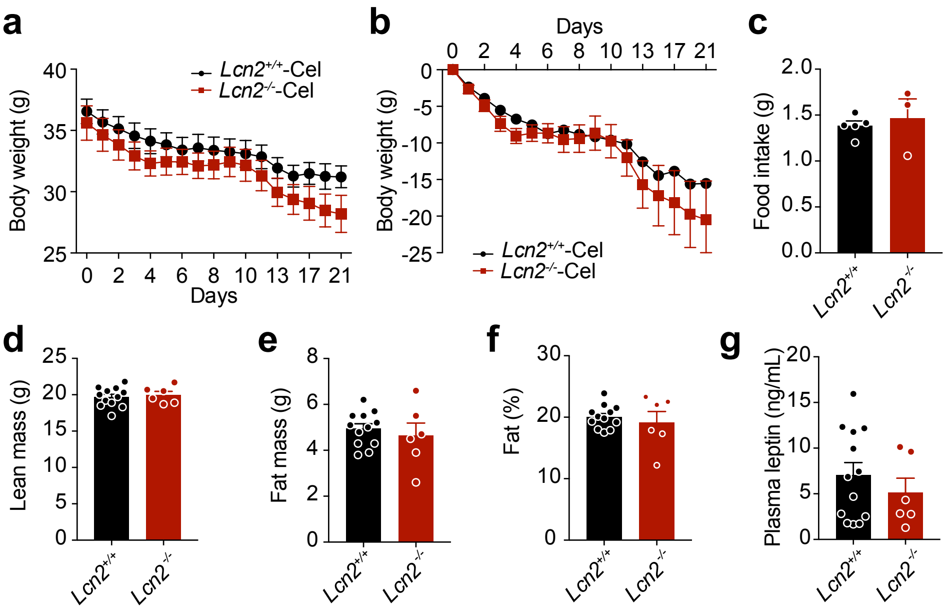

a
Lcn2+/+-Cel
Lcn2-/--Cel
Body weight (g)
40
35
30
25
0 2 4 6 8 10 13 17 21
Days
b
Days
0 2 4 6 8 10 13 17 21
Body weight (g)
0
-5
-10
-15
-20
-25
Lcn2+/+-Cel
Lcn2-/--Cel
c
Food intake (g)
2.0
1.5
1.0
0.5
0.0
Lcn2+/+
Lcn2-/-
d
Lean mass (g)
25
20
15
10
5
0
Lcn2+/+
Lcn2-/-
e
Fat mass (g)
8
6
4
2
0
Lcn2+/+
Lcn2-/-
f
Fat (%)
30
20
10
0
Lcn2+/+
Lcn2-/-
g
Plasma leptin (ng/mL)
20
15
10
5
0
Lcn2+/+
Lcn2-/-

Supplementary Figure 1. Celastrol decreases body weight and food intake of *Lcn2*$^{+/+}$ and *Lcn2*$^{-/-}$ mice. The mice were fed on a HFD for 16 weeks and then acclimated with intraperitoneal injection of vehicle (DMSO) for seven days. Subsequently, both groups of *Lcn2*$^{+/+}$ and *Lcn2*$^{-/-}$ mice were administered with celastrol (100 μg/kg, i.p., once a day) for 3 weeks. (**a**) Body weight and (**b**) percent change in body weight of *Lcn2*$^{+/+}$ mice and *Lcn2*$^{-/-}$ mice during celastrol treatment (*n*=13 for *Lcn2*$^{+/+}$ mice; *n*=7 for *Lcn2*$^{-/-}$ mice). (**c**) Average 24h food intake of *Lcn2*$^{+/+}$ mice and *Lcn2*$^{-/-}$ mice during the first week of celastrol treatment. (**d-f**) Lean mass, fat mass and fat % after 3 weeks of celastrol treatment. (**g**) Plasma leptin levels (ng/ml) in *Lcn2*$^{+/+}$ and *Lcn2*$^{-/-}$ mice after 1 week-celastrol treatment. Values indicate average ± s.e.m. *P* values were determined by two-way ANOVA (a, b) and two-tailed Student *t* test (c-g).

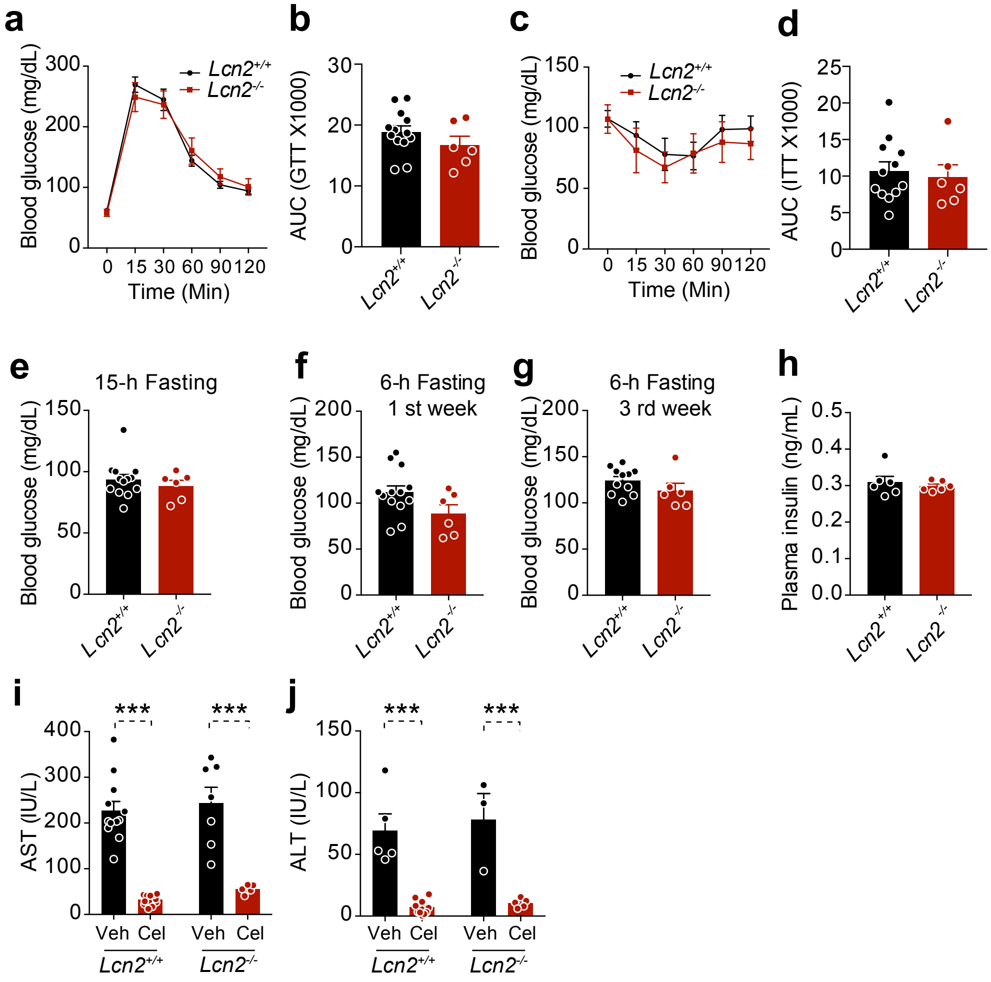
a
b
c
d
e
f
g
h
i
j
Blood glucose (mg/dL)
Time (Min)
AUC (GTT X1000)
AUC (ITT X1000)
Lcn2+/+
Lcn2-/-
15-h Fasting
6-h Fasting
1 st week
3 rd week
Plasma insulin (ng/mL)
AST (IU/L)
ALT (IU/L)
***
Veh
Cel

Supplementary Figure 2. Celastrol improves glucose homeostasis in *Lcn2*$^{+/+}$ and *Lcn2*$^{-/-}$ mice.

The mice were fed on a HFD for 16 weeks and then acclimated with intraperitoneal injection of vehicle (DMSO) for 7 days. Subsequently, both groups of *Lcn2*$^{+/+}$ and *Lcn2*$^{-/-}$ mice were administered with celastrol (100 μg/kg, i.p., once a day) for 3 weeks. (**a**) GTT and (**b**) AUC after 1 weeks of celastrol treatment. (**c**) ITT and (**d**) AUC after 2 weeks of celastrol treatment. (**e**) 15-hour fasting blood glucose after 10 days of celastrol treatment. (**f**) Six-hour fasting blood glucose after 7 days of celastrol treatment. g) Six-hour fasting blood glucose after 3 weeks of celastrol treatment. (**h**) Plasma insulin levels after 3 weeks of celastrol treatment. Blood (**i**) AST and (**j**) ALT levels after 3 weeks of celastrol treatment. Values indicate average ± s.e.m. *P* values were determined by two-way ANOVA (a,c,i and j) and two-tailed Student *t* test (b and d-h). *** $P < 0.001$.

Figure 2 e

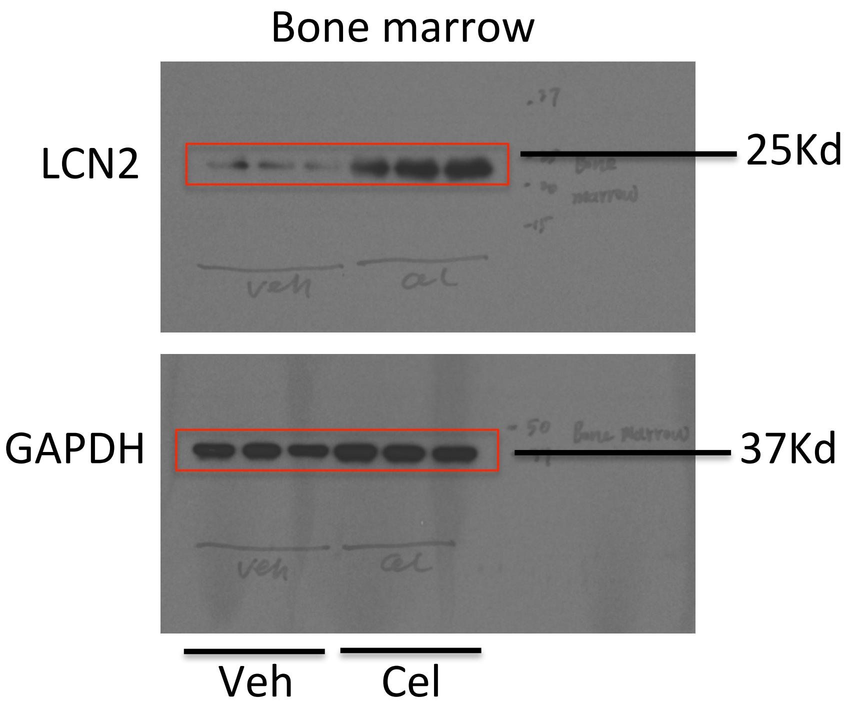

Figure 2 f

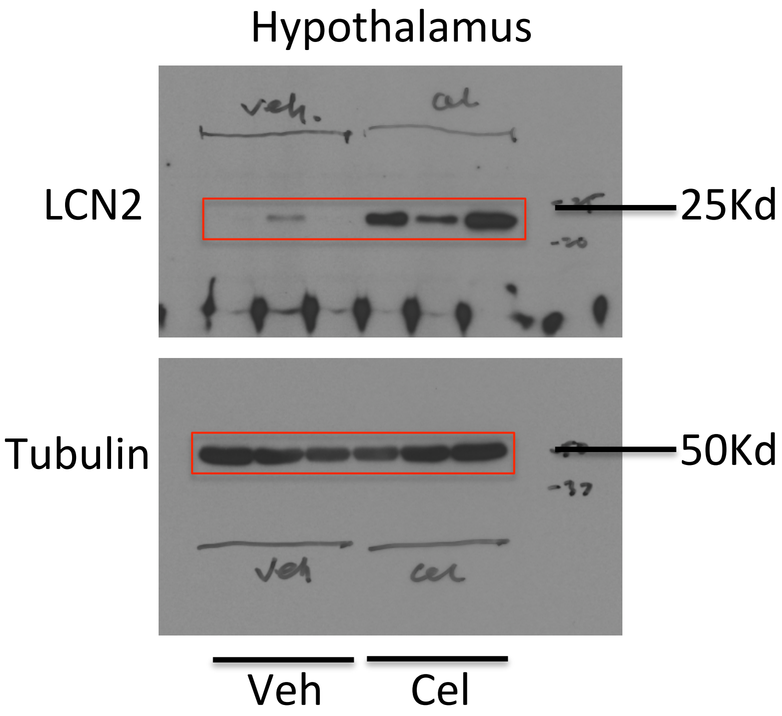

Uncropped blots-01

Figure 2 g

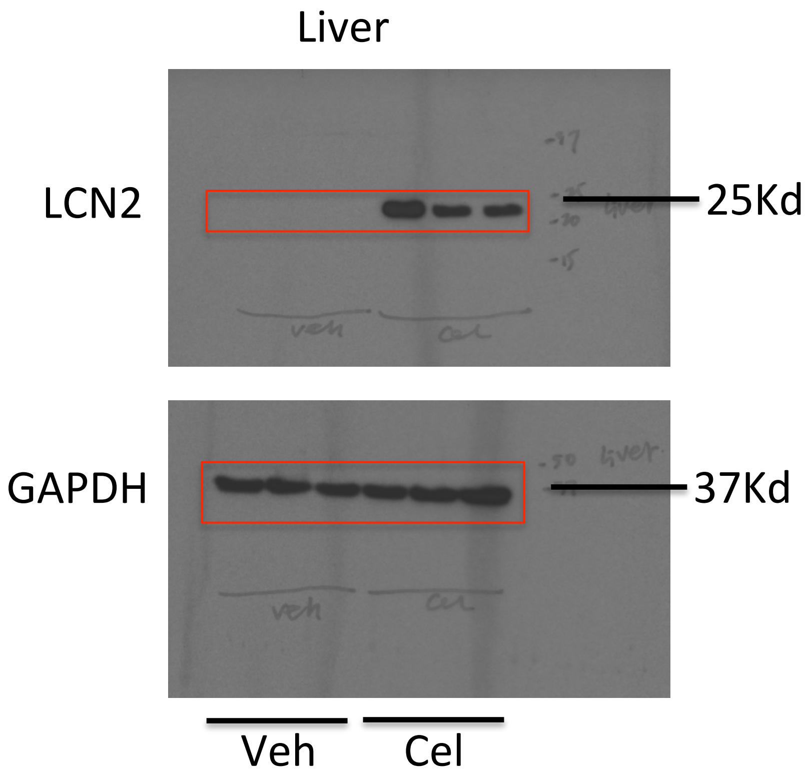

Figure 2 h

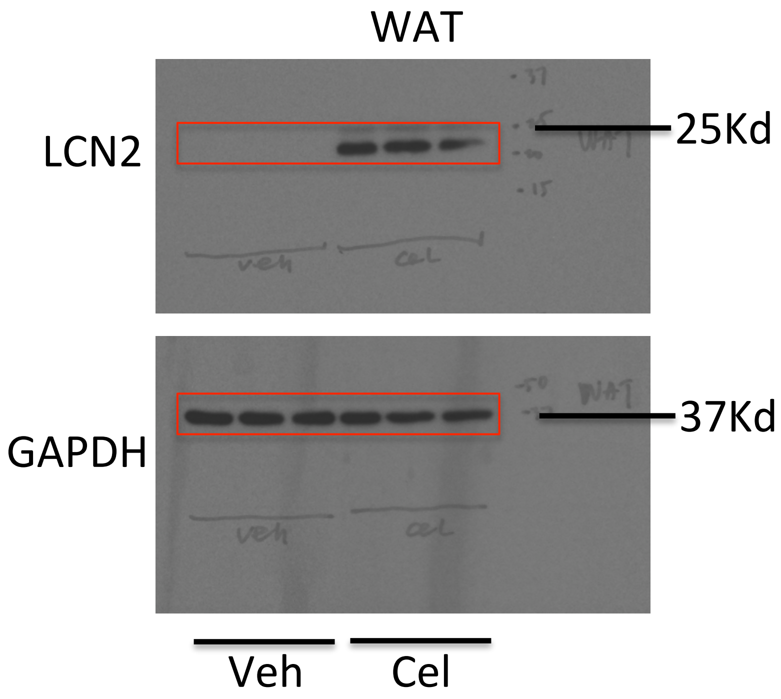

Uncropped blots-02

Figure 2 i

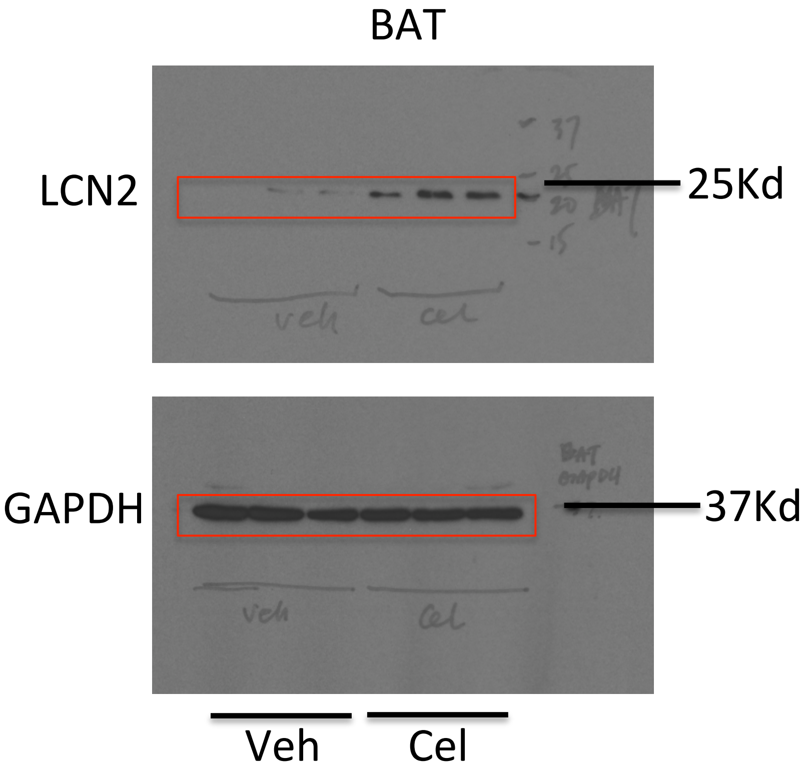

Figure 2 j

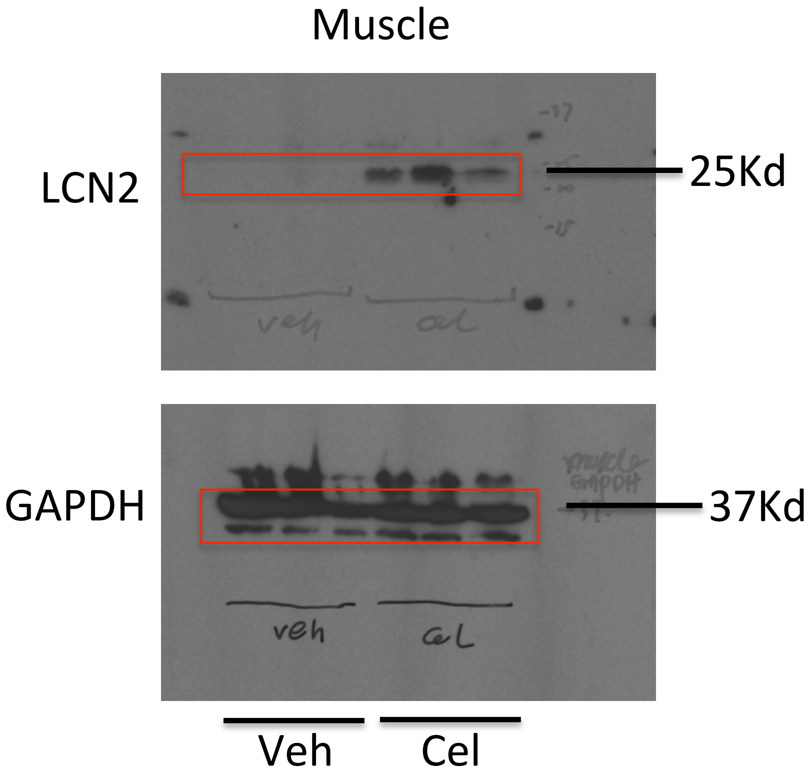

Uncropped blots-03

Figure 3 b

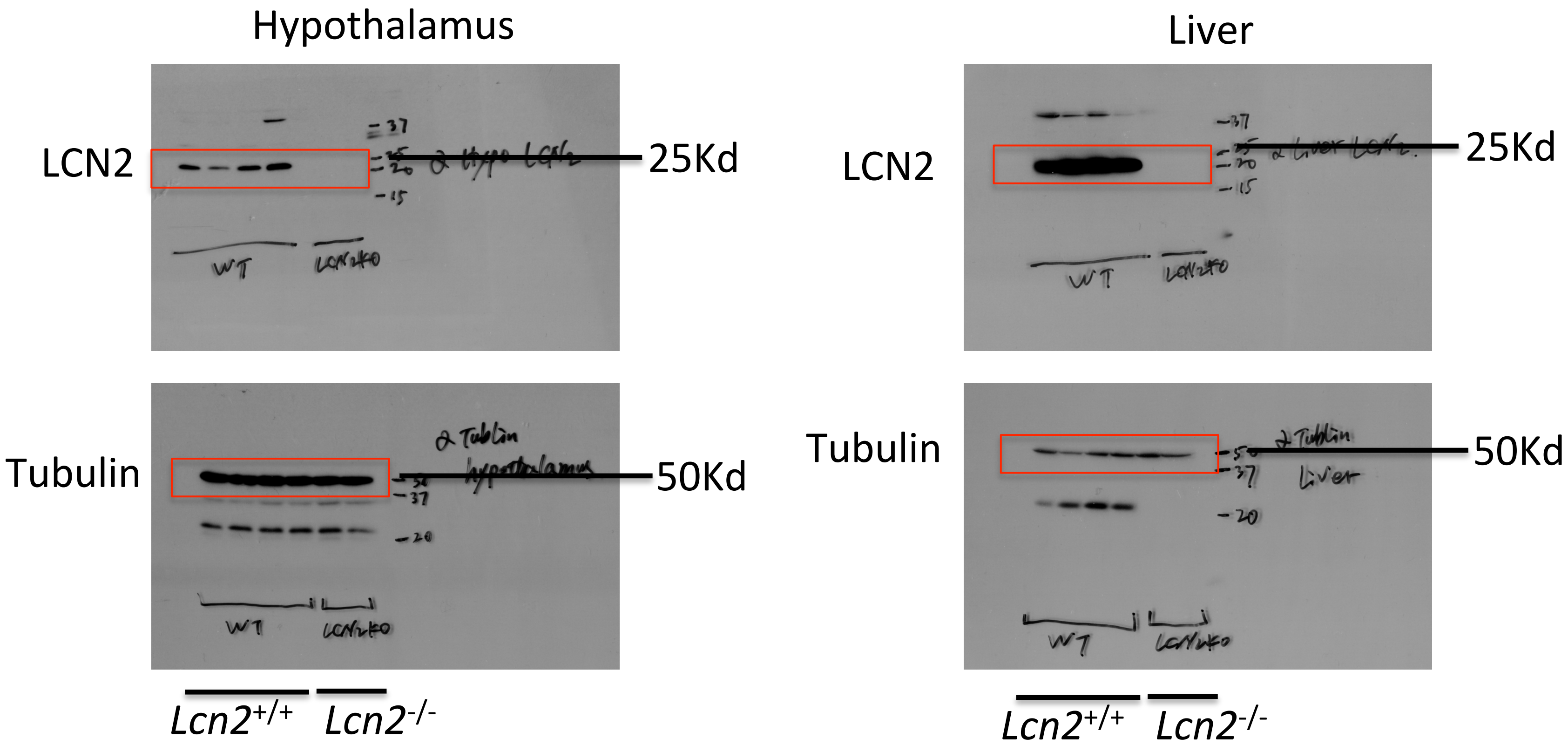

Uncropped blots-04
